# Commentary: the importance of Medicaid expansion for criminal justice populations in the south

**DOI:** 10.1186/s40352-017-0047-0

**Published:** 2017-03-03

**Authors:** Nickolas D. Zaller, David H. Cloud, Lauren Brinkley-Rubinstein, Sarah Martino, Benjamin Bouvier, Brad Brockmann

**Affiliations:** 1 Department of Health Behavior and Health Sciences, University of Arkansas for Medical Sciences, Little Rock, AR USA; 2 Vera Institute of Justice, Substance Use and Mental Health Program, New York City, NY USA; 3 Rollins School of Public Health, Emory University, Atlanta, GA USA; 4 Department of Social Medicine, University of North Carolina, Chapel Hill, NC USA; 5 Center for Health Equity Research, University of North Carolina, Chapel Hill, NC USA; 6 Center for Prisoner Health and Human Rights, Providence, RI USA; 7 Brown University, Providence, RI USA

## Abstract

Though the full implications of a Trump presidency for ongoing health care and criminal justice reform efforts remain uncertain, whatever policy changes are made will be particularly salient for the South, which experiences the highest incarceration rates, highest uninsured rates, and worst health outcomes in the United States. The passage of the Affordable Care Act (ACA) in 2010 was a watershed event and many states have taken advantage of opportunities created by the ACA to expand healthcare coverage to their poorest residents, and to develop partnerships between health and justice systems. Yet to date, only four have taken advantage of the benefits of healthcare reform. Expanding Medicaid would provide Southern states with the opportunity to significantly impact health outcomes for criminal justice-involved individuals. In the context of an uncertain policy landscape, we suggest the use of three strategies, focusing on advancing incremental change while safeguarding existing gains, rebranding Medicaid as a local or statewide initiative, and linking Medicaid expansion to criminal justice reform, in order to implement Medicaid expansion across the South.

## Background

The United States has the highest incarceration rate in the world. People in correctional institutions are significantly less healthy than the general population. Disproportionately high numbers of criminal justice (CJ) involved individuals report chronic physical, mental health, and substance use disorders (SUDs). For instance, national surveys of jails indicate rates of SUDs of 70% and symptoms of mental illness of almost 65% (Karberg and James, 2005; James and Glaze, 2006). In addition, incarcerated individuals have higher rates of diabetes, arthritis, HIV, Hepatitis C, tuberculosis, and sexually transmitted diseases, among other health issues, than the general population (Binswanger et al., 2012). Moreover, these conditions are often diagnosed at more advanced stages in CJ-involved individuals than in similar non-incarcerated populations (Dumont et al., 2012), contributing to poor health outcomes across the life course. Rates of incarceration and poor health are particularly pronounced in the U.S. South.

### The situation in the south

People living in the United States (US) South are incarcerated at a disproportionate rate when compared to individuals in other regions of the US (Carson and Sabol, 2012). Nearly every state in the South has an incarceration rate that continues to exceed the national average, and it is the only region with an incarceration rate above 450 per 100,000 population (Wagner, 2014). Two Southern states have an incarceration rate that is more than twice that figure; Louisiana’s imprisonment rate is the highest in the country (Carson and Sabol, 2012). Importantly, in nine Southern states, more than half of the prison population is black: Alabama, Delaware, Georgia, Louisiana, Maryland, Mississippi, North Carolina, South Carolina, and Virginia. In Maryland, 72% of the prison population is African American (Nellis, 2016). This exacerbates existing health disparities as African Americans are more likely to report fair or poor health status relative to all other racial and ethnic groups in the South (Stephens et al., 2014).

State-level surveillance data on health outcomes among correctional populations is very limited. However, people incarcerated in the South have relatively poorer health than those in other regions because these individuals have less access to health services and more risk factors for morbidity and mortality prior to incarceration. In 2010, there were twice as many people diagnosed with HIV/AIDS in Southern prisons than Northeastern prisons, and five times the number in Western and Midwestern facilities (Maruschak, 2012). Southern prisons also spend comparatively less money per patient providing healthcare services inside prisons, and have higher mortality rates than those in other regions (The Pew Charitable Trusts and the MacArthur Foundation, 2014). Of the total number of state prisoners who died between 2000 and 2012, almost half were incarcerated in the South (Noonan et al., 2015).

### The Affordable Care Act (ACA): challenges and opportunities

The passage of the ACA in 2010 was a watershed event that continues to have an impact on the US healthcare system. The expansion of Medicaid eligibility, though optional for states, is the ACA’s central structural reform for abating healthcare access related inequalities in the US. Historically, Medicaid benefits have only been available to impoverished children and their parents, pregnant women, and individuals with disabilities. The ACA extends Medicaid coverage to young, childless adults with an income up to 138% of the federal poverty level regardless of their personal or health status. To date, 31 states and the District of Columbia have expanded Medicaid under the ACA. However, most Southern states have chosen not to do so.

Exacerbating incarceration’s impact on health is the fact that many incarcerated individuals lack access to affordable insurance both before incarceration and after release. A recent article noted that upon reentry, as many as 90% were uninsured prior to passage of the Affordable Care Act (ACA) (Patel et al., 2014). In the majority of Southern states that have failed to expand Medicaid to date, many formerly incarcerated individuals remain uninsured. In addition, the disparity between disease burden and post-release access to care can drive up the cost of health care, result in worse outcomes, and cause patients to seek care later than appropriate (Patel et al., 2014). Expanding Medicaid coverage to low income individuals is a critical step to addressing these significant public health issues. The US Government Accountability Office (GAO) estimated in September 2014 that the majority of CJ-involved individuals in expansion states would qualify for Medicaid (GAO, 2014). In New York and Colorado, for example, officials found that the *vast* majority (80 and 90%, respectively) would likely qualify. In contrast, in North Carolina, a state that has not expanded Medicaid, officials estimated that only about 2% of CJ-involved individuals were eligible for the program (GAO, 2014). As a result, a perilous coverage gap has emerged for large numbers of CJ-involved individuals in the South with unmet chronic mental and physical health needs.

Many states have taken advantage of opportunities created by the ACA to expand healthcare coverage to their poorest residents, and to develop partnerships between health and justice systems to simultaneously abate health disparities and enhance public safety. People with serious mental illness enrolled into Medicaid are more likely to utilize community health services and are less likely to recidivate than those without health insurance. This suggests the importance of Medicaid expansion to support vulnerable and at-risk individuals as they transition from corrections to the community (Morrissey et al., 2007). And while the future of the ACA remains in doubt, states will likely retain discretion regarding Medicaid expenditures and coverage options even with a full repeal of the ACA. Thus, it is of critical importance that states which have opted to expand Medicaid coverage preserve the gains made in insurance coverage for criminal justice populations, and for those which have opted not to expand Medicaid under the ACA to find opportunities to provide insurance coverage for those in the criminal justice system who remain uninsured.

While limited, research suggests that Medicaid expansion is positively associated with significant improvements in access to care and mortality – particularly among minorities and residents of low-income counties, compared with those in non-expansion states (Nguyen and Sommers, 2016; Sommers et al., 2012; Sommers et al., 2015). Perhaps no single population stands more to gain from expanded Medicaid coverage than CJ-involved individuals with complex health needs. For example, it is estimated that roughly 25–30% of jail detainees could benefit from Medicaid expansion nationally, and one in six new Medicaid enrollees will be CJ-involved (Regenstein and Rosenbaum, 2014). In addition, expanded Medicaid funding can play a critical role in building and establishing treatment-based incarceration diversion programs and promoting continuity of care at release by reimbursing community based providers for delivering services to CJ-involved individuals immediately after release. By increasing the number of people receiving treatment in community settings, particularly behavioral health treatment, states may be able to decrease their prison and jail populations—and their correctional spending.

As of January 2015, 64 programs across 21 states were devoted to enrolling CJ-involved individuals into health insurance and linking them to care. Notably, 89% of these programs were located in Medicaid expansion states, and most programs began functioning only after implementation of the ACA (Bandara et al., 2015). In 2014 alone, the first year of Medicaid expansion, the percentage of CJ-involved individuals with substance use disorders who lacked health insurance dropped from 38 to 28% (Saloner et al., 2016). In addition, a recent analysis of the National Survey on Drug Use and Health data found that in 2014, nationwide, the ACA improved rates of mental health treatment for people with serious psychiatric conditions as well (Ali et al., 2015). Thus, Medicaid expansion can have a significant impact on access to critical health care, especially behavioral health care, among CJ populations. However, expansion of Medicaid in southern states has faced formidable challenges.

## Recommendations

Expanding Medicaid, or affordable healthcare insurance options, provides Southern states with the opportunity to significantly impact health outcomes for CJ-involved individuals. Yet to date, only four have taken advantage of the benefits of healthcare reform. Kentucky, Arkansas, West Virginia, and, as of June 2016, Louisiana, have chosen to expand access to insurance coverage for low-income residents. Drawing on examples provided by these states, we put forth the following recommendations to other Southern states that have not yet expanded Medicaid coverage. These recommendations include the following strategies: 1) Focus on incremental change that might include a “private option” or coverage of only the poorest citizens as a first step; 2) Rebrand Medicaid as a local or statewide initiative; and 3) Link Medicaid expansion to the mission of criminal justice reform.

### Learning from Other Southern States

The two earliest adopters of Medicaid expansion in the South were Kentucky and Arkansas. In 2013, Arkansas adopted Medicaid expansion through what is commonly termed the Private Option, which is the use of Medicaid funds to support enrollment in either Medicaid or private insurance plans procured through the health exchange market place. Arkansas was the first state in the nation to take such an approach to Medicaid expansion. Pre-expansion Arkansas had strict Medicaid eligibility requirements and one in five adults were uninsured. In contrast, at the beginning of 2015, over 230,000 people were eligible to enroll in the private option and over 219,000 had enrolled (Wishner et al., 2015). In addition, other key milestones from Arkansas’s expansion efforts include a reduction in hospital related uncompensated care by 55% and reduction in insurance premiums largely fueled through generating a younger, healthier risk pool (Guyer et al., 2015).

Kentucky also opted to expand Medicaid coverage in 2014 with a specific focus on providing healthcare to the lowest-income individuals in the state (adults with incomes up to 138% of the federal poverty line). During its first 6 months in operation, the expansion program contributed to the cutting the number of uninsured individuals in the state nearly in half. In addition, the uninsured rate for adults fell from 18.8 to 6.8% between 2013 and 2015. This decrease represents one of the largest reductions in the uninsured rate in the US (Cohen and Martinez, 2014; Cohen et al., 2016). Louisiana is the most recent example of a Southern state that has chosen to expand Medicaid. It, like Kentucky, is taking an incremental approach and is only increasing insurance availability to individuals up to 138% of the FPL.

Arkansas provides compelling evidence that evidence that incremental approaches can be successful even in Southern states where there may be political and ideological opposition to Medicaid expansion. Remaining Southern states could use this strategy citing evidence of efficacy from these other Southern states to slowly begin to gain support for healthcare reform.

### Rebrand Medicaid expansion as a local or statewide initiative

Another strategy to move toward Medicaid expansion in Southern states includes rebranding Medicaid expansion as an independent local or statewide “initiative” in order to distance themselves from the politicized rhetoric associated with President Barack Obama (i.e. Obamacare). In 2015, a local poll by the *Arkansas Times* asked readers several questions related to the ACA. When they asked only if the legislature should approve the private option a majority said yes. When the question alluded to the fact that the private option was a component of Obamacare a majority opposed its passage. Subsequently, Arkansas rebranded their program as “Arkansas Works” and was approved during the most recent legislative session for another year. Other states have also rebranded their programs, framing expansion as a state-driven effort rather than a federal mandate. For example, Kentucky’s Medicaid expansion program is called KYNECT and uses a state-based marketplace. Taking ownership of Medicaid expansion through branding efforts, local capacity and infrastructure building can help states to administer expansion on their own terms on their own timeline. This approach may increase acceptance of Medicaid expansion among constituents and legislative bodies alike.

### Linking Medicaid expansion to the mission of criminal justice reform

Government officials in Southern states are increasingly supportive of criminal justice reform as part of a growing conservative movement that recognizes corrections spending has significantly increased without corresponding decreases in recidivism. The conservative approach, as articulated by groups such as Right on Crime, favor addiction and mental health treatment-based diversion programs for people charged with low level crimes as a less expensive alternative to incarceration (Gingrich and Nolan, 2011). However, the success of these treatment-based programs, as well as reentry strategies focused on treatment and recidivism reduction, hinges on the accessibility and quality of those services. Medicaid expansion is a sustainable solution for funding these programs and expanding them to meet high needs.

Policymakers, community based organizations and individuals who understand criminal justice reform’s importance must be strategic in linking Medicaid expansion to long-term cost savings especially for vulnerable populations, including those with CJ-experience. They can look to their peers who have developed creative and effective strategies to provide expanded health coverage to their citizenry despite immense challenges. It is critical that in states where Medicaid has expanded and efforts to enroll CJ-involved individuals are already underway, research is conducted to examine the impact on use of health services among newly enrolled CJ-involved individuals as well as the impact on recidivism. Some evidence has emerged demonstrating that leaders in Southern states are aware of the relationship between CJ reform and Medicaid. A recent statement by Rebekah Gee, Louisiana’s Health Secretary alluded to the importance of “treating mental illness and addiction rather than incarcerating” (O’Donnell, 2016). Similar rhetoric can be used to make the case for Medicaid expansion as an important component of CJ reform. Finally, states seeking to implement criminal justice reform can make Medicaid expansion as a key feature of such reform.

## Conclusion

Given that individuals in Southern states are both incarcerated at a higher rate and experience worse health outcomes compared to other regions of the US, we recommend Medicaid expansion as one possible solution to improve health outcomes among Southerners with CJ-experience. We suggest the use of three strategies, focusing on incremental change, rebranding Medicaid as a local or statewide initiative, and linking Medicaid expansion to CJ reform, in order to implement Medicaid expansion across the South. These strategies have been successful in four Southern states and can be easily replicated across the region focus. However, we acknowledge that while expanded insurance coverage is necessary, it certainly is not sufficient to ensure access to essential medical care among individuals with CJ-experience. There is also an urgent need for guidance regarding best practices for leveraging Medicaid expansion to optimally improve outcomes for formerly incarcerated individuals upon reentry. Finally, given the new political landscape nationally, there must be increased expectation for state leadership to promote states as laboratories for experiments in health care reform. Southern states like Arkansas, Kentucky, Louisiana and West Virginia can be an examples for navigating the new policy landscape.
